# Review: *Dicranostigma leptopodum*: A peculiar plant of *Papaveraceae*

**DOI:** 10.1515/biol-2022-0963

**Published:** 2024-11-22

**Authors:** Ye Tian, Yang Geng, Yaolei Zhang, Zeyue Zhao, Lihong Ruan, Chuanbao Zhang

**Affiliations:** Henan Railway Food Safety Management Engineering Technology Research Center, Department of Pharmacy, Zhengzhou Railway Vocational and Technical College, Zhengzhou, Henan, 451460, China

**Keywords:** *Dicranostigma leptopodum* (*Maxim*.) Fedde, species distribution, chemical constituents, extraction and separation methodologies, content determination, pharmacological activities

## Abstract

*Dicranostigma leptopodum* (*Maxim*.) Fedde, an intriguing plant of the *Papaveraceae* family, exhibits numerous physiological activities, including antitumor, cytotoxic, immune-boosting, antibacterial, antioxidant, insecticidal, smooth muscle modulation, antiarrhythmic, fluorescent probe capabilities, as well as hypolipidemic and hypoglycemic effects. The plant’s diverse pharmacological actions are believed to be attributed to its rich reservoir of alkaloids distributed throughout the entire plant. This review encompasses an exploration of the chemical constituents, pharmacological activities, species distribution, extraction and separation methodologies, and content determination of *D. leptopodum*, aiming to contribute to its further advancement in medicinal development.

## Graphical abstract

**Figure:**
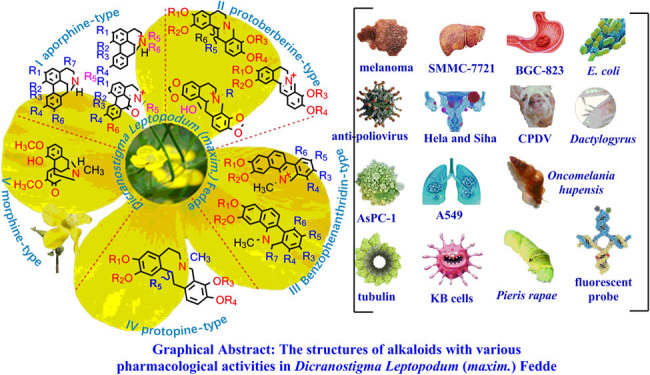

## 1 Introduction

Currently, within the *Dicranostigma Hook. f. et Thoms.* classification, six species have been identified: *Dicranostigma lactucoides Hook. f. et Thoms.* (DLT), *Dicranostigma platycarpum C.Y. Wu et H. Chuang* (DPC), *Dicranostigma leptopodum* (Maxim.) *Fedde* (DLF), *Dicranostigma franchetianum* (*Prain*) Fedde (DFF), *Dicranostigma iliensis C.Y. Wu et H Chuang* (DIW), and *Dicranostigma henanensis S.Y. Wang et L. H. Wu* (DHW) [1]. Among them, DLF or DFF are also known as Yunnan Sichuan tu chuang hua and hong mao cao, respectively [2,3]. These traditional medicinal herbs, derived from roots, possess cooling, bitter, astringent, and toxic properties. They are traditionally associated with heat-clearing, detoxification, reduction of swelling, and pain relief. These herbs find application in treating various conditions such as tonsillitis, toothache, sore throat, scrofula, gingival swelling and pain, scalp favus, scabies, carbuncle, tinea capitis, tinea corporis, and alopecia. Additionally, these herbs can be applied topically for alopecia, scabies, carbuncle, fistula, persistent stomatitis, suppurative otitis media, gastric ulcers, trauma, herpes zoster, scrotal tinea, vulval swelling and pain, mycotic vaginitis, and contagious ovine ecthyma (ORF), demonstrating notable therapeutic effects [4,5].

Compared to other species within the *Dicranostigma* genus, DLF stands out due to its higher concentration of alkaloids, particularly protoberberine derivatives, which are responsible for many of its medicinal properties [6]. Furthermore, recent studies have demonstrated that DLF possesses potent anti-inflammatory and analgesic effects, making it a promising candidate for the development of novel therapeutic agents [7]. Given the significant medicinal value and the unique chemical composition of DLF, this study focuses specifically on this species to explore its potential therapeutic applications and elucidate the mechanisms underlying its biological activities. By concentrating on DLF, we aim to provide a comprehensive understanding of its bioactive constituents and their pharmacological effects, thereby contributing to the development of evidence-based herbal therapies.

## 2 Main chemical constituents in DLF

### 2.1 Alkaloids

According to the Chinese Materia Medica, DLF primarily contains isoquinoline alkaloids. These include corydrine (CORY), isocorydrine (Iso-CORY), corytuberine, glaucine, magnoflorine (MAG), menisperine, dicranostigmine, corydaline, d-isocorydaline, isocorypalmine, sanguinarine (SAN), protopine (PRO), allocryptopine (ALL), and sinoacutine, which are derived from various parts of the plant [8]. For instance, chelerythrine (CHE), chelirubine (CHEL), cryptocavine, SAN, ALL, MAG, and PRO have been isolated from the roots [1]. Additionally, Chelombit’ko [9] collected aerial parts of DFF at the flowering stage and isolated berberine (BER, 12), coptisine (COP, 15), ALL, PRO, and Iso-CORY.

Gregorová et al. [10] isolated six compounds – SAN, CHE, BER, ALL, PRO, and COP – from the ethanol extracts of dried DLF roots using high-performance liquid chromatography (HPLC). In another study by Liu et al. [11], isoquinoline alkaloids were identified from DLF sourced from Pingliang (Gansu). These included 2 benzophenanthridines, 1 morphine, 4 aporphines, and 4 protoalkaloids identified as dihydrosanguinaline (D-SAN), 6-acetonyl-5,6-dihydrosanguinarine, N-methyl-hernovine, *cis*-protopinium, *trans*-protopinium, sinoacutine, dicranostigmine, isocorydaline, CORY, PRO, and ALL, respectively.

Moreover, Sun et al. [5] separated nine alkaloids – 10-O-methylhernovine, nantenine, corytuberine, lagesianine A, CORY, Iso-CORY, D-SAN, dihydrochelerythrine (D-CHE), PRO, and dihydrocryptopine – with three different structural skeletons of aporphine, protoberberine, and PRO by employing repeated silica gel column chromatography (CC).

Additionally, Zhong et al. [8] reported two quaternary protoberberine alkaloids, berberrubine and 5-hydroxycoptisine, from the entire plants of DLF. These alkaloids were isolated from the genus *Dicranostigma* for the first time, and the structure of a new compound was elucidated using various spectroscopic methods.

### 2.2 Triterpenoids

Wang and Li [12] discovered a novel hopane triterpene named Dicranostigmone and identified erythrodiol-3-*O*-palmitate, a previously known compound, in the petroleum ether extract of the 95% ethanol extract derived from the entire DLF plant located in Pinliang city, Gansu province. Additionally, Liu isolated β-sitosterol and β-daucosterin (β-sitosterol-3-*O*-glucoside) for the first time from the same DLF plant extract [13].

The structures of alkaloids and triterpenoids extracted from DLF are presented in Table 1.

**Table 1:** Structures of alkaloids and triterpenoids from DLF

Structure type	No.	Compound structure	Compound name (Abb.)	Molecular formula	Molecular weight	Plant parts [references]
I Aporphine-type alkaloids 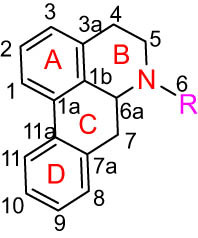	1	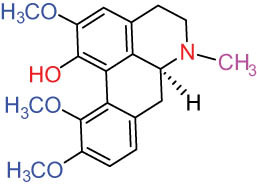	(+)-Corydrine (CORY)	C_20_H_23_NO_4_	341.4	Whole plant [1,5,8,12]
	2	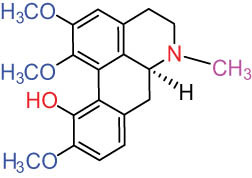	(+)-Isocorydrine (Iso-CORY)	C_20_H_23_NO_4_	341.4	Whole plant [1,5,8,12]; aerial parts [8]
	3	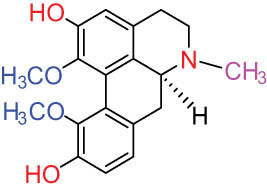	(+)-*N*-methylhernovine	C_19_H_21_NO_4_	327.4	Whole plant [8,12]
	4	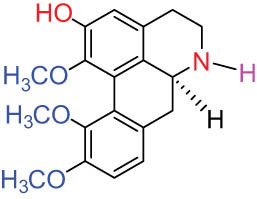	10-*O*-methylhernovine	C_19_H_21_NO_4_	327.4	Whole plant [12]
	5	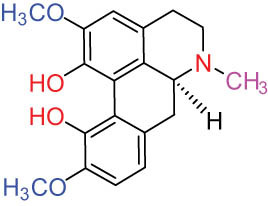	Corytuberine	C_19_H_21_NO_4_	327.4	Whole plant [1,5]
	6	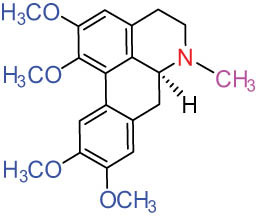	Glaucine	C_21_H_25_NO_4_	355.4	Whole plant [1,5]
	7	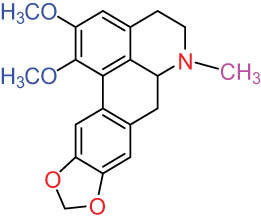	Nantenine	C_20_H_21_NO_4_	339.4	Whole plant [12]
	8	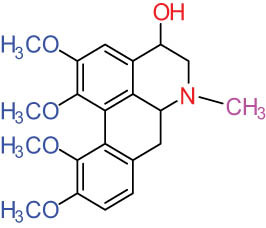	Lagesianine A	C_20_H_23_NO_5_	371.4	Whole plant [12]
	9	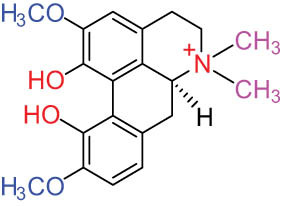	Magnoflorine (MAG)	C_20_H_24_NO_4_^+^	342.5	Whole plant [1,5]; roots [8,8]
	10	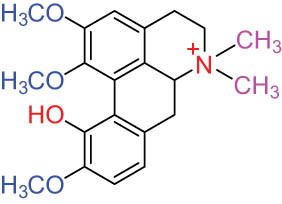	Menisperine	C_21_H_26_NO_4_^+^	356.4	Whole plant [1,5]
	11	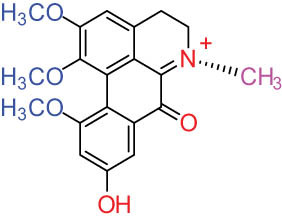	Dicranostigmine	C_20_H_20_NO_5_	354.1	Whole plant [1,5,14]
II Protoberberine-type alkaloids 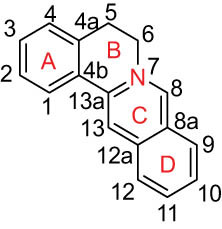	12	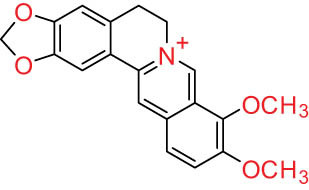	Berberine (BER)	C_20_H_18_NO_4_^+^	235.3	Aerial parts [10]; roots [11]
	13	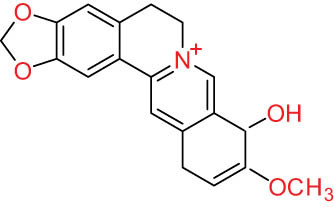	Berberrubine	C_19_H_16_NO_4_^+^	322.1	Whole plant [14,20]
	14	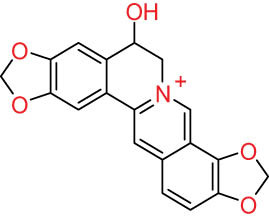	5-Hydroxycoptisine	C_19_H_14_NO_5_^+^	336.1	Whole plant [14,20]
	15	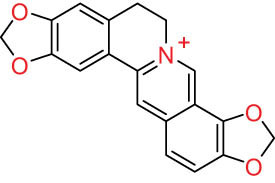	Coptisine (COP)	C_19_H_14_NO_4_	320.3	Aerial parts [10]; roots [11]
	16	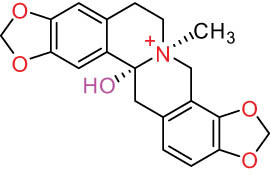	*cis*-Protopinium	C_20_H_20_NO_5_	354.1	Whole plant [8]
	17	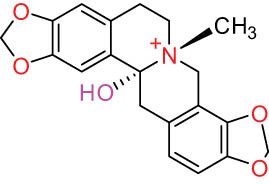	*trans*-Protopinium	C_20_H_20_NO_5_	354.1	Whole plant [8]
	18	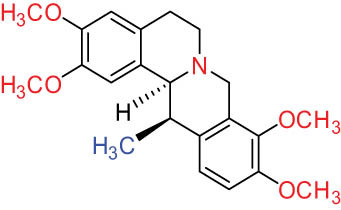	Corydaline	C_22_H_27_NO_4_	369.4	Whole plant [1,5]
	19	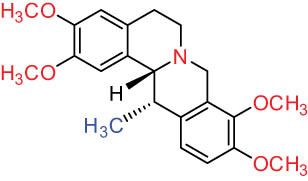	d-Isocorydaline	C_22_H_27_NO_4_	369.4	Whole plant [1,5]
	20	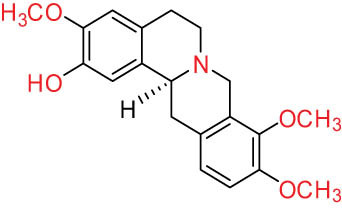	(−)-Isocorypalmine	C_20_H_23_NO_4_	341.4	Whole plant [1,5]
III Benzophenanthridine-type alkaloids 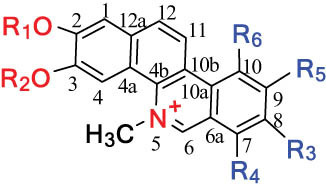 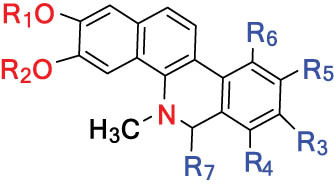	21	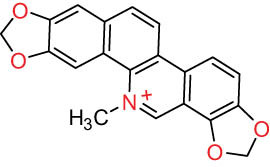	Sanguinarine (SAN)	C_20_H_14_NO_4_^+^	332.3	Whole plant [1,5]; roots [8,9]
	22	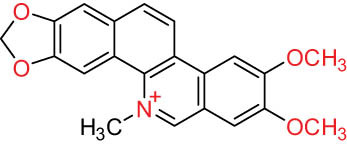	Chelerythrine (CHE)	C_21_H_18_NO_4_^+^	348.4	Roots [8,9,11]
	23	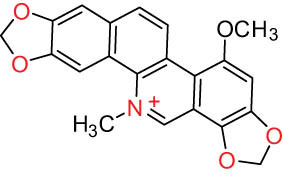	Chelirubine (CHEL)	C_21_H_16_NO_5_^+^	362.3	Roots [8,9]
	24	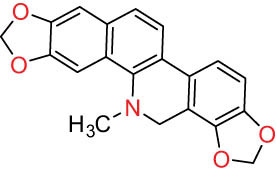	Dihydrosanguinaline (D-SAN)	C_20_H_15_NO_4_	333.3	Whole plant [8,12]
	25	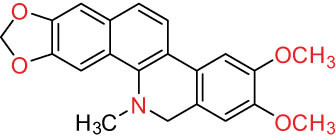	Dihydrochelerythrine (D-CHE)	C_21_H_19_NO_4_	349.4	Whole plant [12]
	26	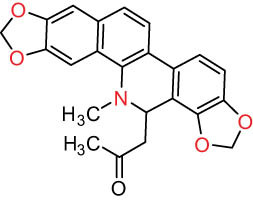	6-Acetonyl-5,6-dihydrosanguinaline	C_23_H_19_NO_5_	389.4	Whole plant [8]
IV Protopine-type alkaloids 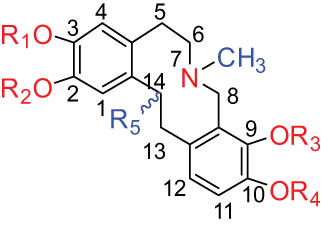	27	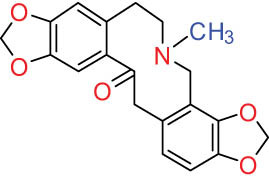	Protopine (PRO)	C_20_H_19_NO_5_	353.3	Whole plant [1,5,8,12]; roots [8,9,11]; aerial parts [10]
	28	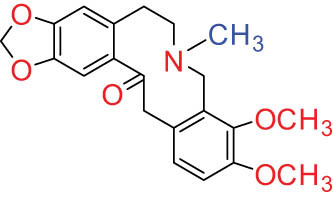	Allocryptovine (ALL)	C_21_H_23_NO_5_	369.4	Whole plant [1,5,8]; roots [7,8,11]; aerial parts [10]
	29	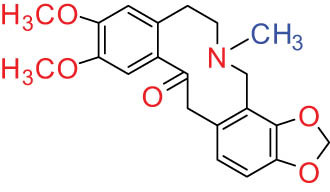	Cryptocavine	C_21_H_23_NO_5_	369.4	Roots [8,9]
	30	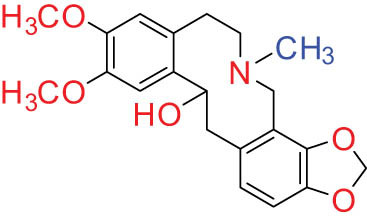	Dihydrocryptopine	C_21_H_25_NO_5_	371.4	Whole plant [12]
Ⅴ Morphine-type alkaloids 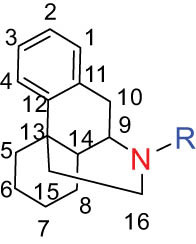 morphinane	31	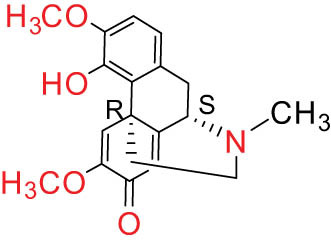	Sinoacutine	C_19_H_21_NO_4_	327.4	Whole plant [1,5,8]
Ⅵ Triterpenes 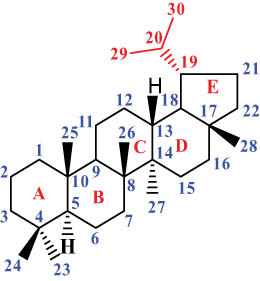 Pentacyclic triterpenoids	32	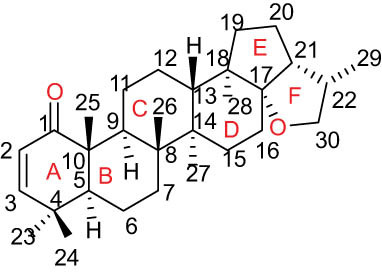 lupine-type	Dicranostigmone	C_30_H_46_O_2_	439.4	Whole plant [12]
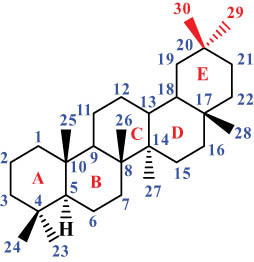 Pentacyclic triterpenoids	33	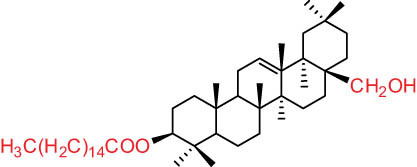 oleanane-type	Erythrodiol-3-*O*-palmitate	C_46_H_80_O_3_	681.1	Whole plant [12]
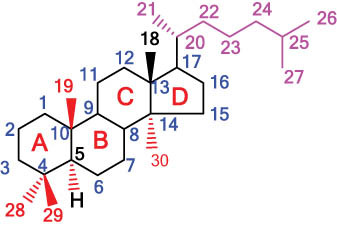 Tetracyclic triterpenoids	34	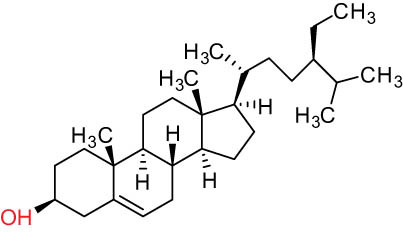 lanostane-type: lanosterol	β-Sitosterol	C_29_H_50_O	414.7	Whole plant [14]
Tetracyclic triterpenoids: lanostane-type: triterpenoid saponin	35	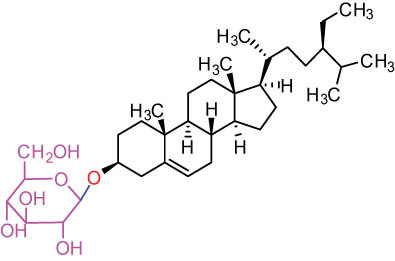	β-Daucosterine	C_35_H_60_O_6_	576.9	Whole plant [14]

## 3 Extraction and separation of active sites and components from DLF

### 3.1 Ethanol extraction, acid-soluble alkali precipitation, chloroform extraction, silica gel CC

Initially, dried DLF was pulverized and subjected to extraction using 95% ethanol. The ethanol solvent was then removed through decompression, and the resulting extract was reconstituted in a 2% H_2_SO_4_ aqueous solution. Subsequently, an acidified chloroform extract was derived via chloroform extraction, concentrating the resulting extractant under reduced pressure. The pH of the aqueous solution was adjusted to 9 using ammonia water, followed by an extraction process using chloroform to acquire the alkalized chloroform phase. This alkalized chloroform extract underwent gradient elution on a silica gel CC using a chloroform–methanol–triethylamine solvent system [14].

### 3.2 Process of ethanol ultrasound extraction-Al_2_O_3_ CC

The hay powder of DLF was soaked or macerated with 95% ethanol and sealed for 2–3 days at room temperature, typically around 25°C. Following this, ultrasonic vibration was applied for 1 h, and the resultant mixture was then filtered under normal pressure. The filtrate obtained was concentrated under reduced pressure until no alcoholic taste was perceived. The ethanol extract obtained underwent elution with chloroform using a dry neutral Al_2_O_3_ (200–300 mesh) quartz CC. Subsequently, the chloroform eluate was concentrated under vacuum. NaOH was then added to the concentrated eluate, and the mixture was allowed to stand for 2–3 days. The resulting white crystals formed were collected through vacuum filtration and subsequently dried to yield the final product [15].

### 3.3 Process of preparation of dicranostigmine by high-pressure chromatography

Dicranostigmine was synthesized from the ethanol extract of DLF utilizing medium-pressure (ranging from 50 to 20 bar) preparative chromatography. The specific chromatography conditions employed were as follows: the QuikSep-50 medium-high pressure chromatography system was utilized along with a C18SMB100-20/45 (5 μm) filler within a self-loading high-pressure stainless steel pipe column measuring 30 mm × 250 mm. The column was maintained at a pressure of 2 MPa throughout the process. The mobile phase used was a mixture of methanol and water in a ratio of 80:20. Detection of the compound was conducted at a wavelength of 328 nm, with the column temperature set at room temperature. The flow rate during the process was maintained at 14 mL/min, and the injection volume was 4 mL. The purity of the synthesized sample was evaluated as 88%, calculated based on the normalization of the peak obtained via HPLC [8].

### 3.4 Simultaneous determination of alkaloids in DLF by HPLC

The purification process of the effective fraction of alkaloids (EFA) in DLF involved using D101 macroporous resin. High-performance liquid chromatography with diode-array detection (HPLC-DAD) was established to determine the total alkaloid content of EFA. Results indicated that the total alkaloid content of EFA was 19.04 ± 1.23% as determined by the HPLC-DAD method. Meanwhile, UV-visible spectrophotometry suggested the total alkaloid content of EFA to be 65.92 ± 1.33%. These newly established methods demonstrated rapid and accurate determinations with high repeatability, paving the way for further applications of EFA from DLF [13].

Furthermore, a straightforward and efficient method was developed for the simultaneous determination and effective fractionation of eight isoquinoline alkaloids in the methanol extracts of DLF using HPLC-DAD. The chromatographic conditions were optimized on a SinoChrom ODS-BP column to identify two aporphines (Iso-CORY and CORY), two protopines (PRO and ALLO), morphine (sinoacutine), and three protoberberine alkaloids (BER, 5-hydroxycoptisine, and berberrubine). The optimized chromatogram conditions for the standard solution included wavelengths at 270 and 360 nm, mobile phase consisting of acetonitrile (A) and 0.2% phosphoric acid in water adjusted to pH 6.32 by triethylamine solution (B), gradient elution from 20 to 26% (A) at 0–20 min, 26–50% (A) at 20–35 min, holding at 50% (A) at 35–37 min, 50–20% (A) at 37–40 min, and finally 20% (A) at 40–45 min. The injection volume was 20 μL, and the flow rate was 1.0 mL/min [4].

## 4 Pharmacological effects of DLF

The crude extracts and active constituents derived from DLF showcase a diverse range of pharmacological activities. These activities encompass antitumor and cytotoxic properties, immune protection, antimicrobial effects against pathogens, antioxidant capabilities, insecticidal attributes, impacts on smooth muscle function, anti-arrhythmic potential, utilization as a DNA fluorescence probe, as well as hypolipidemic and hypoglycemic activities.

### 4.1 Antitumor and cytotoxic activities

Hammerová et al. [15] discovered that SAN, CHE, and CHEL extracted from DLT exhibited potent anti-proliferative effects on melanoma cells. These compounds also demonstrated efficacy in treating malignant melanoma, including p53 deficient melanoma, by inducing apoptosis through reduced levels of anti-apoptotic proteins (Bcl-xL, Mcl-1, XIAP), mitochondrial membrane potential disruption, and activation of caspase-3 and poly ADP-ribose polymerase cleavage [16]. SAN displayed antitumor effects in epithelial ovarian cancer cells by modulating the CASC2-EIF4A3 axis, inhibiting NF-κB signaling or the PI3K/AKT/mTOR pathway [17], and by upregulating Fas-associated factor 1 in non-small cell lung cancer [18]. Liposomal SAN demonstrated dose-dependent cytotoxic activity (CTA) against prostate cancer cell lines LNCaP, DU 145, and PC-3, inducing efficient apoptosis in both hormone-sensitive and -independent cells. Thus, liposomal SAN emerges as a promising antitumor agent for both hormone-sensitive and -independent tumors [19].

Additionally, Fox et al. [20] identified PRO, ALL, and cryptocavine from DLF as topoisomerase I toxicants, resembling camptothecins for their cytotoxicity in human cancer cell cultures. Sun et al. [5] isolated nine alkaloids with diverse structural skeletons (aporphine, protoberberine, and PRO) from DLF. Cell proliferation and cytotoxicity assays using the cell counting kit-8 demonstrated that these alkaloids hinder the growth of human lung cancer cells (H1299), breast cancer cells (MCF-7), and human hepatoma cells (SMMC-7721) via CTA. Furthermore, nantenine, corytuberine, and D-SAN exhibited cytotoxic effects on the SMMC-7721 cell line both *in vitro* and *in vivo*. Lagesianine A exhibited anti-poliovirus activity. Although CORY and Iso-CORY were inactive against KB cells, they curbed the proliferative activity of hepatocellular carcinoma cell lines by inducing G2/M stage cell cycle arrest [21] and apoptosis related to programmed cell death 4 [22]. D-SAN exhibited superior CTA compared to PRO against human non-small cell lung cancer cells (A549), human colon cancer cells (HT-29), oral cancer cells (KB cells), and mouse leukemia cells (P-388) [23]. Consequently, a series of mild C(sp3)-H functionalization of D-SAN and D-CHE were synthesized to develop highly cytotoxic derivatives against human breast, colorectal, and prostate cancer cell lines, exhibiting IC_50_ values in the range of 0.6–8.2 μM, akin to the positive control doxorubicin. D-SAN and D-CHE represent potential lead compounds for the development of novel anticancer agents [24]. The aforementioned studies highlight the greater cytotoxicity of BER-type alkaloids compared to PRO and aporphine. The structure–activity correlation revealed the critical role of 1-OH and 11-OH of aporphine alkaloids in the cytotoxicity on selected cell lines, while 1-OCH3 did not exhibit any cytotoxicity. These compounds might target the D2 receptor in cells. Nevertheless, further research is indispensable to elucidate the mechanism of DLF alkaloids.

Moreover, Ahsan et al. [25] demonstrated that SAN triggers apoptosis in human pancreatic cancer cells, AsPC-1 and BxPC-3, by modulating the B-cell lymphoma-2 (Bcl-2) protein. SAN’s induction of apoptosis and cell cycle disturbance attributes its effects to the modulation of Bcl-2 and the human tumor suppressor gene (p53). SAN hindered viability, growth, and colony formation ability, induced apoptosis and G0–G1 phase cell cycle arrest, elevated pro-apoptotic Bax, Bid, and Bak proteins while reducing anti-apoptotic Bcl-2 and Bcl-X(L) proteins. It also decreased p53 levels but enhanced its phosphorylation in AsPC-1 and BXPC-3 cells in a dose-dependent manner. This study suggests SAN’s potential as a therapeutic agent for managing pancreatic cancer. Nonetheless, further studies employing animal models are imperative. The mechanisms involved encompass increased levels of apoptotic proteins (Bax, Bid, and Bak), reduced inhibition of anti-apoptotic proteins (Bcl-2 and Bcl-XL), and increased phosphorylation yet decreased levels of the human tumor suppressor gene p53. Thus, SAN holds promise as a therapeutic agent for pancreatic cancer.

Wolff and Knipling [26] presented evidence that benzophenanthridine alkaloids, namely CHEL, SAN, and CHE, act as inhibitors, impeding paclitaxel-mediated microtubulin aggregation in rat brains. CHEL competitively weakens the binding of colchicine to tubulin without affecting podophyllotoxin binding to microtubulin. Conversely, SAN inhibits the binding of colchicine and podophyllotoxin to microtubulin. The IC_50_ values of SAN were measured at 32 and 46 mM, respectively, while those of CHE were 55 and 60 mM, respectively. Notably, the combined effect of the two drugs was synergistic. The disappearance of fluorescence peaks at 596 nm and yellow staining indicated the potential reversible formation of an acid-pseudobase by SAN via tubulin, involving several sulfhydryl groups and its ammonium ion. Additionally, the pharmacological impact of these drugs might be due to their role in hindering tubulin polymerization and preventing cell mitosis.

Overall, aporphine-type, benzophenanthridine-type, and protoberberine-type alkaloids found in DLF potentially constitute the fundamental framework of leading compounds for antitumor drugs.

### 4.2 Immune protection

The therapeutic options for Severe Acute Respiratory Syndrome Coronavirus (SARS-CoV-2) are currently confined to repurposed drugs aimed at symptom management and nonspecific interventions to bolster the human immune system. This study employed chromatographic and *in silico* methodologies to pinpoint bioactive compounds potentially acting as inhibitors for SARS-CoV-2, alongside human immunomodulators like tumor growth factor-beta (TGF-β) and tumor necrosis factor-alpha (TNF-α). Notably, bioactive compounds such as BER and MAG were identified as potent inhibitors against the main protease (Mpro) of SARS-CoV-2 through multiple docking strategies. These inhibitors demonstrated significant roles in viral replication/transcription during infection and targeted the conserved binding cleft common among various coronavirus strains [27].

MAG was observed to notably upregulate TNF-α and interleukin-1 beta levels, prostaglandin E2 production, and cyclooxygenase-2 protein expression. This, in turn, stimulated mRNA transcription levels of these pro-inflammatory mediators and enhanced NF-κB activation by triggering phosphorylation of p65, IκBα (inhibitor of NF-κB), and IKK (inhibitor of kappa B kinase) α/β. Additionally, it prompted IκBα degradation and upregulated MyD88 (myeloid differentiation primary response gene 88) and TLR4 (Toll-like receptor 4). These results suggested that MAG amplifies immune responses, contributing to immunomodulation [28]. Shan demonstrated that DLF induced a significant O_2_ effect in peritoneal macrophages (PMΦ) in mice. DLF intake positively affected PMΦ activity and phagocytosis, along with increased lysozyme levels, showcasing the activation of PMΦ and enhancement of immune function [29].

Moreover, experiments conducted by Zhang et al. [30] in a hypoimmune mouse model indicated that DLF injection activated PMΦ, significantly enhancing immune function in mice with low immune responses. The injection also notably improved nonspecific immunity and specific humoral immunity, as reflected by alterations in various measured indicators. In an immune-related liver injury model disrupted by Bacillus Calmette-Guerin (BCG) and lipopolysaccharide (LPS) in mice, DLF administration resulted in lowered levels of liver enzymes (AST, ALT, LDH), reduced malondialdehyde (MDA) content in liver homogenate, and alleviated liver tissue damage compared to the control group. These findings suggest that the DLF extract offers protective effects against immune-related liver injury induced by BCG and LPS [31].

Additionally, Zhang [32] reported significantly reduced levels of liver injury markers and increased superoxide dismutase (SOD) levels in mice treated with different DLF doses compared to the CCl4-induced acute chemical liver injury group. Microscopic pathology revealed DLF’s protective effect against hepatocyte degeneration and necrosis caused by CCl4-induced liver injury, potentially attributed to its anti-free radical effect and involvement in nonspecific immune defense [33].

In summary, various DLF extracts or their natural components, including MAG, exhibit potential as immunomodulators contributing to immune protection in the domain of Chinese medicine.

### 4.3 Anti-pathogenic microorganism action

Li [34] utilized the plate dilution method and determined that the minimum inhibitory concentration (MIC) values of the 95% ethanol extract of DLF against Gram-positive and Gram-negative bacteria were 0.18 mg/mL for *Escherichia coli*, 0.14 mg/mL for *Staphylococcus aureus*, and 0.26 mg/mL for *Enterococcus faecalis*, highlighting the notable antibacterial effect of DLF.

Xue [35] established that DLF preparations effectively hinder the proliferation of the H52 strain (infectious bronchitis virus) in chicken embryos. The effective concentration ranged from 2.5 to 5 mg/mL, and its inhibitory effect was positively correlated with increasing DLF concentration.

Chen et al. [36] presented the MIC values of β-magarine against various fungi, showcasing values of 10 μg/disc for *Penicillium avellaneum* (UC-4376), 100 μg/disc for *Pyricularia oryzae*, and 5 μg/disc for *Penicillium corylus*. Notably, no inhibition of *Magnaporthe oryzae* was observed at 100 μg/disc.

Wei et al. [37] evaluated the inhibitory effects of CHE against *Ustilaginoidea virens* and *Cochliobolus miyabeanus* with 50% effective concentrations (EC50) of 6.53 × 10^−3^ mg/mL and 5.62 × 10^−3^ mg/mL, respectively. Additionally, CHE exhibited high efficacy in inhibiting spore growth of *U. virens* and outperformed the commercial fungicide validamycin. After treatment with CHE, significant morphological changes occurred in *U. virens* mycelia, indicating potential apoptosis induction in pathogenic fungi.

Cheng and Liu [38] demonstrated the robust inhibitory effects of various alkaloids (MAG, SAN, CHE, and ALL) in DLF against the acid production of *Streptococcus mutans*. The observed bacteriostatic and antibacterial effects are attributed to the combined activity of these alkaloids.

Qin and Dang [39] screened the medicinal components of DLF by water-soluble injection of murine interferon-gamma (mIFN-γ) and determined the standard for the DLF water-soluble preparation that contains 250 mg/mL of dry substance. The killing rate of 10^6^ *E. coli* was >95% at 4°C after 72 h, and the average nitrogen content was 2.29%. The average content of IFN-γ of 5 × 10^5^ leukomonocytes was >450 pg/mL, and the average nitrogen content was 1.67% *in vitro* culture medium of 1-month-old Balb/c mice. The bactericidal effect of the water extract from the DLF water-soluble preparation was significantly higher than the precipitation of the water extract and exhibited adequate antibacterial and immunomodulatory functions.

MAG exhibited substantial antifungal activity against *Trichophyton rubrum* and *Trichophyton mentagrophyte*, demonstrating better fungicidal efficiency against *T. rubrum* than *T. mentagrophyte*. Its impact on *T. rubrum* encompassed inhibiting conidia germination, suppressing hyphal growth, and inducing morphological alterations in the mycelium, including deformation growth, surface peeling, and cytoplasmic contraction. While MAG did not significantly affect cell wall integrity, it effectively disrupted the fungal cell membrane of *T. rubrum*. This disruption led to increased nucleic acid leakage, reduced activities of squalene epoxidase and CYP51 enzyme, and decreased ergosterol content in hyphae, all indicative of its potential as an effective antifungal agent [40]. Isoquinoline alkaloids, including BER, COP, corydaline, CHEL, D-SAN, CHE, and SAN, exhibited varying degrees of inhibition against plant fungi [41]. Among these, SAN displayed notable antifungal activity within a range of 6.96–59.36 μg/mL, exhibiting superior inhibitory effects against *M. oryzae* when compared to azoxystrobin. SAN significantly suppressed spore germination of *M. oryzae*, achieving a 100% inhibition rate at 50 μg/mL. Observations through optical microscopy and scanning electron microscopy following treatment of *M. oryzae mycelia* with SAN at 10 μg/mL revealed substantial alterations in mycelial structure, including curvature, collapse, and damaged cell membrane integrity. Additionally, changes were observed in reactive oxygen species production, mitochondrial membrane potential, and nuclear morphometry, indicating the disruption of membrane function and cell proliferation of the mycelium. These findings provide valuable insights into the mechanisms underlying SAN’s potent antifungal activity against *M. oryzae*.

### 4.4 Antioxidant activities

Zhao et al. investigated the impact and mechanism of DLF extract on H_2_O_2_-induced oxidative hemolysis of mouse erythrocytes *in vitro* and acetylphenylhydrazine (APH) *in vivo*. Consequently, the H_2_O_2_-induced oxidative hemolysis was suppressed, leading to increased erythrocyte count and hemoglobin content. Additionally, the activity of catalase (CAT), glutathione peroxidase (GSH-Px), and glucose-6-phosphate dehydrogenase (G-6-PD) was enhanced, while SOD activity remained unaltered. These findings indicated that DLF extract mitigated H_2_O_2_- or APH-induced hemolysis, potentially attributed to increased G-6-PD activity, consequently alleviating oxidative hemolysis of erythrocytes by inhibiting APH interference with G-6-PD [42].

Moreover, Aihong examined the effect of DLF on hepatic microsome injury resulting from lipid peroxidation after CCl4 administration in mice. They assessed the activities of SOD, CAT, and GSH-Px in liver plasma, and Na^+^, K^+^-ATPase, and Ca^2+^-ATPase in hepatic microsomes suspension using commercial kits. They also measured the activities of NADPH and cytochrome C (P450) reductase in the microsome via spectrometry. Additionally, the levels of MDA in hepatic microsome suspension were measured using the thiobarbituric acid method. The results demonstrated that DLF pretreatment significantly increased the activities of SOD and GSH-Px, notably enhancing Na^+^, K^+^-ATPase, and Ca^2+^-ATPase activities, and partially mitigating the rise in NADPH cytochrome C (P450) reductase activity and MDA levels. These findings indicated that DLF stimulated hepatic antioxidant enzyme activity, neutralizing free radicals generated by CCl4 metabolism, thus exhibiting a protective effect against resulting liver injury in mice [43].

### 4.5 Insecticidal activities

Wang [44] demonstrated SAN’s robust killing activity against *Dactylogyrus* in goldfish water, with a lethal range between 0.4 and 0.6 mg/L. It exhibited a complete lethal effect on *Dactylogyrus* at a 100% insecticidal rate beyond 0.6 mg/L, while its toxic effect on goldfish exceeded 1 mg/L, indicating a higher tolerance threshold.

Ming [45] employed the indoor immersion method to create specific SAN concentrations. *Oncomelania hupensis*, immersed for 24, 48, 72, and 96 h at a constant temperature of 25°C, exhibited mortality rates of over 90% at 25 mg/L for 48 h, 6.25 mg/L for 72 h, and 0.78125 mg/L for 96 h. A complete mortality rate was observed at 25 mg/L for 72 h and 12.5 mg/L for 96 h. The findings revealed SAN’s pronounced lethality against *O. hupensis*, even at low mass concentrations.

Zhou and Yang [46] evaluated the insecticidal activity of ALLO through the insect immersion method. Their bioassay results displayed substantial bioactivity of ALLO against *Pieris rapae* larvae, *Tribolium castaeum* adults, and *Culex quinquefasciatus* larvae. The mortality rates of *C. quinquefasciatus* 3rd instar larvae were 70.00, 73.33, 80.00, and 86.67% at 0.05 mg/mL over 12, 24, 48, and 72 h, respectively. Non-selective rejections of *P. rapae* 5th instar larvae were 84.03 and 89.76% at 0.5 mg/mL in 24 and 48 h, respectively. *T. castaeum* adults exhibited oviposition contraceptive rates of 56.67, 70.00, 76.67, and 83.33% at 0.5 mg/mL over 12, 24, 48, and 72 h, respectively. These experimental findings highlight SAN and ALLO as integral components of DLF insecticides.

## 5 Effects on smooth muscle

Li et al. [47] investigated the indirect and direct effects of PRO on the intracellular calcium concentration ([Ca^2+^]*i*) in rat vascular smooth muscle cells (VSMCs) via the Ca^2+^-free Ca^2+^ method and fluorescence calcium indicator (Fura-2/AM). Their study revealed that 10, 30, and 100 μmol/L PRO significantly inhibited the transient contraction of NA-induced vascular strips in Ca^2+^-free Krebs solution in a concentration-dependent manner. Moreover, PRO dose-dependently reduced the persistent contraction induced by NA after Ca^2+^ restoration. In VSMCs loaded with Fura-2/AM, PRO (at 50 and 100 μmol/L) did not significantly affect [Ca^2+^]*i* at rest. However, in the presence of exogenous calcium, PRO was observed to mitigate the increase in [Ca^2+^]*i* triggered by NA and high K^+^ levels. This suggested that PRO might impede vascular smooth muscle contraction by suppressing Ca^2+^ release and/or influx in response to NA and high K^+^ stimulation, contributing to its vasodilator effect.

In a prior investigation on rabbit aorta, PRO’s vasodilatory effect was linked to increased cyclic adenosine monophosphate (cAMP) and cyclic guanosine monophosphate (cGMP) [48]. Moreover, PRO attenuated NA and high K^+^-induced aortic strip contractions and significantly reduced the maximal response. In Fura-2/AM-loaded VSMCs, PRO (at 50 and 100 μmol/L) did not induce notable changes in resting [Ca^2+^]*i* levels but significantly mitigated the elevation of [Ca^2+^]*i* caused by NA and high K^+^. Additionally, PRO (at 30 and 100 μmol/L) led to the translocation of protein kinase C (PKC) from the cytosol to the membrane in the presence of NA, indicating its potential role in modulating PKC translocation, [Ca^2+^]*i* reduction, and effects on cAMP and cGMP levels, contributing to its vasodilatory effect.

Fan [49] identified PRO as a potential candidate for anti-asthma treatment. They reported that PRO exhibited pD_2_’ *in vitro* values of 3.11 and 3.45 for constrictions induced by histamine and acetylcholine, respectively. Moreover, in rabbit tracheal smooth muscle cells (TSMCs), PRO demonstrated an increase in cAMP levels, while no significant change was observed in cGMP levels. Interestingly, PRO inhibited cAMP-PDE (phosphodiesterase) activity in guinea pig TSMCs without showing a significant effect on cGMP-PDE activity. Additionally, PRO successfully inhibited TSM constriction. These findings suggest that one of the mechanisms underlying PRO’s action might involve its increased impact on cAMP levels via the inhibition of cyclic nucleotide PDE activity in the trachea, leading to the relaxation of tracheal smooth muscle.

### 5.1 Antiarrhythmic effects

Lu [50] conducted a study utilizing eight animal models to investigate the antiarrhythmic effects of PRO. Their findings revealed that PRO demonstrated protective effects against arrhythmia induced by aconitine, calcium chloride, and benzene adrenaline in rats. Additionally, it showed effectiveness in preventing ventricular fibrillation induced by chloroform (CHCl_3_) in mice and ventricular fibrillation triggered by electrical stimulation in rabbits. The proposed underlying mechanism of this effect involved the blockage of the Na^+^ channel in myocardial cells, which led to a prolonged refractory period.

### 5.2 Fluorescence properties or DNA fluorescence probe

The study conducted by Cao [51] focused on exploring the fluorescence and absorption spectra of MAG. They found that in the aqueous solution of MAG, three distinct fluorescent peaks appeared in the three-dimensional (3D) fluorescence spectra. These peaks were observed at excitation wavelengths [*λ*(ex)] of 230, 275, and 315 nm, emitting at the same wavelength [*λ*(ex)] of 420 nm. Notably, variations in pH levels of the solution caused shifts in the fluorescence peak and led to the emergence of an isosbestic point in the absorption spectrum, indicating proton ionization in a hydroxyl group within the MAG molecule. Through the pH-absorption method, they determined the proton ionization constant (p*K*_a_ = 4.77) of MAG.

Using l-tryptophan as a reference, the researchers calculated the fluorescence quantum yield (*Y*) of the aqueous MAG solution to be 0.19. These characteristic fluorescence peaks were identified in the 3D fluorescence spectra of various Chinese herbal medicines. By preparing extracts of *Sinomenium acutum* in a methanol–water solvent, specifically with 60% methanol, the team obtained 3D fluorescence spectra. In the neutral aqueous solution derived from *S. acutum*, the fluorescence peak appeared at *λ*(ex)/*λ*(em) = 315 nm/420 nm, which remained unaffected by coexisting components. Consequently, they established a method for determining MAG in *S. acutum*. The developed regression equation for fluorescence intensity (IF) and MAG concentration (*c*) in the range of 0.04–1.25 μg/mL was IF = 6146.8*c* + 24.4 (*R* = 0.999, *n* = 11), with a detection limit of 0.52 ng/mL. The determined MAG content in *S. acutum* reference material was 0.63%, with a recovery rate of 101.2–102.7%. The fluorescence method was consistent with the LC-MS/MS method, which determined the MAG content in the same sample as 0.61%.

Regarding Rajecky et al.’s study [52], they highlighted that extracts from the Himalayan herb DLT containing CHEL could serve as DNA fluorescent probes. These probes exhibited blue (free-form) and red (intercalated to DNA) luminescence after irradiation by near-UV light. Besides quantifying DNA (with a limit of detection = 6 ng/mL), CHEL showed potential as a supravital cell probe due to its ability to permeate cell membranes.

### 5.3 Hypolipidemic and hypoglycemic activity

The study conducted by Hung et al. [53] demonstrated several notable effects of MAG on lipid peroxidation and its potential in preventing oxidation-related damage. MAG showed protection against human high-density lipoprotein (HDL) from lipid peroxidation. It notably inhibited Cu^2+^-induced HDL lipid peroxidation, prolonging the lag phase of oxidation (62–123 min) at a concentration of 3 mM. Additionally, MAG inhibited the generation of thiobarbituric acid reactive substances in a dose-dependent manner, with IC_50_ values of 2.3 ± 0.2 and 6.2 ± 0.5 mM when HDL oxidation was induced by Cu^2+^ or 2,2-azobis(2-amidinopropane) dihydrochloride, respectively. Moreover, the inclusion of MAG in Cu^2+^-oxidized HDL protected against low-density lipoprotein oxidation, suggesting a preventive role for MAG in HDL oxidation.

Patel and Mishra [54] identified MAG as a promising α-glucosidase inhibitor, indicating its potential as an antidiabetic agent. The enzyme kinetics study using sucrose and maltose as substrates revealed that MAG caused an increase in the apparent Michaelis–Menten constant (Km) and acted as a reversible, competitive inhibitor. The IC_50_ values for MAG as a sucrase inhibitor and a maltase inhibitor were determined to be 9.8 and 7.6 μg/mL, respectively. *In vivo* studies conducted on rats through an oral glucose tolerance test showed that MAG at a dose of 20 mg/kg body weight significantly suppressed (*P* < 0.01) the increase in plasma glucose levels, indicating its potential as an α-glucosidase inhibitor *in vitro* and *in vivo*.

These findings suggest that MAG possesses antioxidative properties against lipid peroxidation, particularly in HDL protection, and exhibits inhibitory effects on α-glucosidase, showcasing potential as an antidiabetic agent.

### 5.4 Analgesic effect

The currently utilized opioid analgesics act as agonists to the mu-opioid receptor (MOR) but are also associated with significant side effects. Hence, medicinal plants serve as crucial sources for potential new drug candidates; morphine and its semisynthetic analogs stand as well-known examples of analgesic medications. As novel MOR agonists, two naturally occurring plant alkaloids, CORY and corydaline, elicit antinociceptive effects in mice upon subcutaneous administration through a MOR-dependent mechanism. Furthermore, CORY and corydaline have been identified as G protein-biased agonists targeting the MOR, not inducing β-arrestin2 recruitment upon receptor activation. These novel scaffolds thus offer promising initial points for future chemical optimization, paving the way for the development of new opioid analgesics with potentially enhanced therapeutic profiles [55].

## 6 Application prospects

The alkaloids present in DLF exhibit a wide array of physiological activities encompassing cytotoxicity, antitumor, antibacterial, antiviral, antioxidation, antiarrhythmic, immunoregulatory, insecticidal, and fluorescence properties within the realm of natural medicine. Numerous isoquinoline alkaloids, such as SAN, CHE, MAG, Cop, Iso-CORY, and corydaline, could potentially account for the anticancer effects attributed to DLF. Three distinct structural skeletons – aporphine, protoberberine, and PRO – might serve as natural precursors for the development of novel drugs. Therefore, there is a necessity to delve into their chemical components, elaborate on the pharmacodynamic basis, and identify the specific antitumor or precursor compounds. This exploration should encompass understanding the chemical foundation responsible for antioxidation and liver protection, as well as isolating antibacterial compounds with substantial efficacy.

## References

[1] Suchomelová J, Bochoráková H, Paulová H, Musil P, Táborská E. HPLC quantification of seven quaternary benzo[c]phenanthridine alkaloids in six species of the family Papaveraceae. J Pharm Biomed Anal. 2007 May;44(1):283–7. 10.1016/j.jpba.2007.02.005, Epub 2007 Feb 13. PMID: 17367981. 17367981

[2] Wang L, Li F, Wang N, Gao Y, Liu K, Zhang G, et al. Characterization of the Dicranostigma leptopodum chloroplast genome and comparative analysis within subfamily Papaveroideae. BMC Genomics. 2022 Dec;23(1):794. 10.1186/s12864-022-09049-8, PMID: 36460956; PMCID: PMC9717546. PMC9717546 36460956

[3] Wijaya V, Janďourek O, Křoustková J, Hradiská-Breiterová K, Korábečný J, Sobolová K, et al. Alkaloids of Dicranostigma franchetianum (Papaveraceae) and berberine derivatives as a new class of antimycobacterial agents. Biomolecules. 2022 Jun;12(6):844. 10.3390/biom12060844. PMID: 35740968; PMCID: PMC9221290. 10.3390/biom12060844 PMC9221290 35740968

[4] Chen Y, Li M, Liu J, Yan Q, Zhong M, Liu J, et al. Simultaneous determination of the content of isoquinoline alkaloids in Dicranostigma leptopodum (Maxim) Fedde and the effective fractionation of the alkaloids by high-performance liquid chromatography with diode array detection. J Sep Sci. 2015 Jan;38(1):9–17. 10.1002/jssc.201400905, Epub 2014 Nov 19. PMID: 25330407. 25330407

[5] Sun R, Jiang H, Zhang W, Yang K, Wang C, Fan L, et al. Cytotoxicity of aporphine, protoberberine, and protopine alkaloids from Dicranostigma leptopodum (maxim.) Fedde. Evid Based Complement Altern Med. 2014;2014:580483. 10.1155/2014/580483, Epub 2014 May 21. PMID: 24963327; PMCID: PMC4055583. PMC4055583 24963327

[6] Wang L, Li F, Wang N, Gao Y, Liu K, Zhang G, et al. Characterization of the Dicranostigma leptopodum chloroplast genome and comparative analysis within subfamily Papaveroideae. BMC Genomics. 2022;23(1):794. 10.1186/s12864-022-09049-8 PMC9717546 36460956

[7] Lei W, Zhu H, Cao M, Zhang F, Lai Q, Lu S, et al. From genomics to metabolomics: deciphering sanguinarine biosynthesis in Dicranostigma leptopodum. Int J Biol Macromol. 2024;257(Pt 2):128727. 10.1016/j.ijbiomac.2023.128727 38092109

[8] Zhong M, Ma YX, Liu JX, Di DL. A new quaternary protoberberine alkaloid isolated from Dicranostigma leptopodum (Maxim) Fedde. Nat Prod Res. 2014;28(8):507–10. 10.1080/14786419.2013.879586, Epub 2014 Feb 5. PMID: 24499388. 24499388

[9] Chelombit’Ko VA. Dicranostigma franchetianum (Prain) Fedde: a plant promising as a source of the alkaloid isocorydine. Pharm Chem J. 1979;13(8):844–5.

[10] Gregorová J, Babica J, Marek R, Paulová H, Táborská E, Dostál J. Extractions of isoquinoline alkaloids with butanol and octanol. Fitoterapia. 2010 Sep;81(6):565–8. 10.1016/j.fitote.2010.01.020, Epub 2010 Feb 1. PMID: 20117181. 20117181

[11] Liu D, Zhang T, Liu J. Chemical constituents of alkaloids from Dicranostigma leptopodum. Chin Herb Med. 2011;42:1505–8.

[12] Wang F, Li YM. New hopane triterpene from Dicranostigma leptopodum (Maxim) Fedde. J Asian Nat Prod Res. 2010 Jan;12(1):94–7. 10.1080/10286020903443028, PMID: 20390749. 20390749

[13] Liu D. Chemical constituents of Dicranostigma leptopodum (Maxim) Fedde. Chin Tradit Herb Drugs. 2011. 10.1007/s10570-010-9464-0.

[14] Sun R, Jiang H, Zhang W, Yang K, Wang C, Fan L, He Q, et al. Cytotoxicity of aporphine, protoberberine, and protopine alkaloids from Dicranostigma leptopodum (Maxim.) Fedde. Evid Based Complement Altern Med. 2014;2014:580483. 10.1155/2014/580483, Epub 2014 May 21. PMID: 24963327; PMCID: PMC4055583. PMC4055583 24963327

[15] Hammerová J, Uldrijan S, Táborská E, Slaninová I. Benzo[c]phenanthridine alkaloids exhibit strong anti-proliferative activity in malignant melanoma cells regardless of their p53 status. J Dermatol Sci. 2011 Apr;62(1):22–35. 10.1016/j.jdermsci.2011.01.006, Epub 2011 Jan 25. PMID: 21324654. 21324654

[16] Chen Y. Total content determination for the effective fraction of the alkaloids in Dicranostigma leptopodum (Maxim.) Fedde by HPLC and ultraviolet-visible spectrophotometry. Anal Methods. 2016;8(12):2645–52.

[17] Zhang S, Leng T, Zhang Q, Zhao Q, Nie X, Yang L. Sanguinarine inhibits epithelial ovarian cancer development via regulating long non-coding RNA CASC2-EIF4A3 axis and/or inhibiting NF-κB signaling or PI3K/AKT/mTOR pathway. Biomed Pharmacother. 2018 Jun;102:302–8. 10.1016/j.biopha.2018.03.071, Epub 2018 Mar 22. PMID: 29571014. 29571014

[18] Wei G, Xu Y, Peng T, Yan J, Wang Z, Sun Z. Sanguinarine exhibits antitumor activity via up-regulation of Fas-associated factor 1 in non-small cell lung cancer. J Biochem Mol Toxicol. 2017 Aug;31(8):2017. 10.1002/jbt.21914, Epub 2017 Mar 14. PMID: 28296008. 28296008

[19] Feldman NB. Preparation of liposomal sanguinarine and study of its cytotoxic effects against prostate cancer cells. Nanobiotechnol Rep (Online). 2020;15(2):230–5.

[20] Fox BM, Xiao X, Antony S, Kohlhagen G, Pommier Y, Staker BL, et al. Design, synthesis, and biological evaluation of cytotoxic 11-alkenylindenoisoquinoline topoisomerase I inhibitors and indenoisoquinoline-camptothecin hybrids. J Med Chem. 2003 Jul;46(15):3275–82. 10.1021/jm0300476, PMID: 12852757. 12852757

[21] Chen L, Tian H, Li M, Ge C, Zhao F, Zhang L, et al. Derivate isocorydine inhibits cell proliferation in hepatocellular carcinoma cell lines by inducing G2/M cell cycle arrest and apoptosis. Tumour Biol. 2016 May;37(5):5951–61. 10.1007/s13277-015-4362-6, Epub 2015 Nov 23. PMID: 26596832. 26596832

[22] Duan X, Li W, Hu P, Jiang B, Yang J, Zhou L, et al. MicroRNA-183-5p contributes to malignant progression through targeting PDCD4 in human hepatocellular carcinoma. Biosci Rep. 2020 Oct;40(10):BSR20201761. 10.1042/BSR20201761, PMID: 33078826; PMCID: PMC7601345. PMC7601345 33078826

[23] Cao Z, Zhu S, Xue Z, Zhang F, Zhang L, Zhang Y, et al. Isoquinoline alkaloids from Hylomecon japonica and their potential anti-breast cancer activities. Phytochemistry. 2022 Oct;202:113321. 10.1016/j.phytochem.2022.113321, Epub 2022 Jul 31. PMID: 35921889. 35921889

[24] Romo-Pérez A, Miranda LD, Chávez-Blanco AD, Dueñas-González A, Camacho-Corona MDR, Acosta-Huerta A, et al. Mild C(sp3)-H functionalization of dihydrosanguinarine and dihydrochelerythrine for development of highly cytotoxic derivatives. Eur J Med Chem. 2017 Sep;138:1–12. 10.1016/j.ejmech.2017.06.021, Epub 2017 Jun 13. PMID: 28641156. 28641156

[25] Ahsan H, Reagan-Shaw S, Breur J, Ahmad N. Sanguinarine induces apoptosis of human pancreatic carcinoma AsPC-1 and BxPC-3 cells via modulations in Bcl-2 family proteins. Cancer Lett. 2007 May;249(2):198–208. 10.1016/j.canlet.2006.08.018, Epub 2006 Sep 26. PMID: 17005319. 17005319

[26] Wolff J, Knipling L. Antimicrotubule properties of benzophenanthridine alkaloids. Biochemistry. 1993 Dec;32(48):13334–9. 10.1021/bi00211a047, PMID: 7902132. 7902132

[27] Manne M, Goudar G, Varikasuvu SR, Khetagoudar MC, Kanipakam H, Natarajan P, et al. Cordifolioside: potent inhibitor against Mpro of SARS-CoV-2 and immunomodulatory through human TGF-β and TNF-α. 3 Biotech. 2021 Mar;11(3):136. 10.1007/s13205-021-02685-z, Epub 2021 Feb 22. PMID: 33643762; PMCID: PMC7898013. PMC7898013 33643762

[28] Haque MA, Jantan I, Harikrishnan H, Abdul Wahab SM. Magnoflorine enhances LPS-activated pro-inflammatory responses via MyD88-dependent pathways in U937 macrophages. Planta Med. 2018 Nov;84(17):1255–64. 10.1055/a-0637-9936, Epub 2018 Jun 15. PMID: 29906814. 29906814

[29] Shan CZ. Influences of Dicranostigma leptopodum (Maxim) Fedde (DLF) on the immune functions of murine peritoneal macrophages (PMΦ). Shanghai J Immunol. 2001;4(21):216–8.

[30] Zhang XW, He LL, Qin W. Enhanced effect of Dicranostigma leptopodum (Maxim.) Fedde on experimental immunosuppression in mice. J Lanzhou Univ (Nat Sci). 2006;42:60–2.

[31] Mao AH. Protective effect of Dicranostigma leptopodum (Maxim.) Fedde on immunological live injury in mice. Chin Pharmacol Bull. 2004;20(8):940–3.

[32] Zhang Y. The protective effect of the effective components of Dicranostigma leptopodum (Maxim.) Fedde on CCl4 liver injury in mice. J Qinghai Med Coll. 2004;1(25):7–11.

[33] Wen JH, Li DY, Liang S, Yang C, Tang JX, Liu HF. Macrophage autophagy in macrophage polarization, chronic inflammation and organ fibrosis. Front Immunol. 2022 Oct;13:946832. 10.3389/fimmu.2022.946832, PMID: 36275654; PMCID: PMC9583253. PMC9583253 36275654

[34] Li TL. Determination on the contents of Dicranostigma leptodum (Maxim.) Fedde alkaloid by TLC and test on its antimicrobial activities. J Tradit Chin Vet Med. 2010;29:47–51.

[35] Xue Z. Observation on the inhibitory effect of injection of Dicranostigma leptopodum (Maxim.) Fedde on infectious bronchitis virus. Gansu Anim Husb Vet. 2004;34:16–7.

[36] Chen JH, Du ZZ, Shen YM, Yang YP. Aporphine alkaloids from Clematis parviloba and their antifungal activity. Arch Pharm Res. 2009 Jan;32(1):3–5. 10.1007/s12272-009-1111-7, Epub 2009 Jan 29. PMID: 19183870. 19183870

[37] Wei QH, Cui DZ, Liu XF, Chai YY, Zhao N, Wang JY, et al. In vitro antifungal activity and possible mechanisms of action of chelerythrine. Pestic Biochem Physiol. 2020 Mar;164:140–8. 10.1016/j.pestbp.2020.01.007. Epub 2020 Jan 28. PMID: 32284120. 32284120

[38] Cheng RX, Liu S. Inhibitory effects of Chelerythrine on the acidogenicity of Streptococcus mutans. J Pract Dent. 2008;24:364–6.

[39] Qin D, Dang Y. Screening test of water soluble preparations of Dicranostigma leptopodum (Maxim.) Fedde. Agric Sci Technol Inf. 2012;21:29–31.

[40] Luo N, Jin L, Yang C, Zhu Y, Ye X, Li X, et al. Antifungal activity and potential mechanism of magnoflorine against Trichophyton rubrum. J Antibiot (Tokyo). 2021 Mar;74(3):206–14. 10.1038/s41429-020-00380-4, Epub 2020 Oct 20. PMID: 33082529. 33082529

[41] Zhao ZM, Shang XF, Lawoe RK, Liu YQ, Zhou R, Sun Y, et al. Anti-phytopathogenic activity and the possible mechanisms of action of isoquinoline alkaloid sanguinarine. Pestic Biochem Physiol. 2019 Sep;159:51–8. 10.1016/j.pestbp.2019.05.015, Epub 2019 May 25. PMID: 31400784. 31400784

[42] Zhao Q, Han Y, Du YP. The effect of Dicranostigma leptopodum (Maxim) fedde (DLF) extraction on suppressing oxidative hemolysis of erythrocytes and its mechanism. J Lanzhou Univ. 2006;32:40–5.

[43] Aihong M. Protection of Picranostigma leptopodum extraction against acute hepatic microsome injuries induced by carbon tetrachloride. Pharmacol Clin Chin Mater Medica. 2011;27:73–5.

[44] Wang G. The application of sanguinarine in the preparation of aquatic animal drugs and the preparation method of its preparation. 2008.

[45] Ming L. The effect of molluscicidal oncomelania hupensis and impact on the MDA content for SAN of Eomecon chionantha Hance. J Cent South Univ For Technol. 2009;29:142–5.

[46] Zhou GZ, Yang MS. Measurement of active ingredient of Macleya cordata and its bioactivity against insects. J Anhui Agric Univ. 2009.

[47] Li B, Wu Q, Shi JS, Sun AS, Huang XN. Effects of protopine on intracellular calcium and the PKC activity of rat aorta smooth muscle. Sheng Li Xue Bao. 2005 Apr;57(2):240–6. PMID: 15830111. 15830111

[48] Yu LM. Vasodilative mechanism of protopine in rabbit aorta. Chin J Pharmacol Toxicol. 2000;14(1):21–5.

[49] Fan JS. Effects of protopine on tracheal smooth muscle and its mechanism. Chin J Pharmacol Toxicol. 2004;18(4):285–8.

[50] Lu Z. Effects of protopine on experimental arrhythmia. Chin Pharm J. 1995;2(30):81–4.

[51] Cao JJ. Fluorescence properties of magnoflorine and its application in analysis of traditional Chinese medicine. Chin J Anal Chem. 2019;47(6):950–6.

[52] Rajecky M, Slaninova I, Mokrisova P, Urbanova J, Palkovsky M, Taborska E, et al. Alkaloid chelirubine and DNA: blue and red luminescence. Talanta. 2013 Feb;105:317–9. 10.1016/j.talanta.2012.10.045, Epub 2012 Nov 2. PMID: 23598024. 23598024

[53] Hung TM, Lee JP, Min BS, Choi JS, Na M, Zhang X, et al. Magnoflorine from Coptidis Rhizoma protects high density lipoprotein during oxidant stress. Biol Pharm Bull. 2007 Jun;30(6):1157–60. 10.1248/bpb.30.1157. PMID: 17541173. 17541173

[54] Patel MB, Mishra SM. Magnoflorine from Tinospora cordifolia stem inhibits α-glucosidase and is antiglycemic in rats. J Funct Foods. 2012;4(1):79–86.

[55] Kaserer T, Steinacher T, Kainhofer R, Erli F, Sturm S, Waltenberger B, et al. Identification and characterization of plant-derived alkaloids, corydine and corydaline, as novel mu opioid receptor agonists. Sci Rep. 2020 Aug;10(1):13804. 10.1038/s41598-020-70493-1, PMID: 32796875; PMCID: PMC7427800. PMC7427800 32796875

